# Tulobuterol inhibits rhinovirus infection in primary cultures of human tracheal epithelial cells

**DOI:** 10.1002/phy2.41

**Published:** 2013-08-22

**Authors:** Mutsuo Yamaya, Hidekazu Nishimura, Lusamba Nadine, Hiroshi Kubo, Nagatomi Ryoichi

**Affiliations:** 1 Department of Advanced Preventive Medicine for Infectious Disease, Tohoku University Graduate School of MedicineSendai, Japan; 2 Virus Research Center, Clinical Research Division, Sendai National HospitalSendai, Japan; 3 Medicine and Science in Sports and Exercise, Tohoku University Graduate School of MedicineSendai, Japan

**Keywords:** Acidic endosomes, human tracheal epithelial cells, rhinovirus, tulobuterol

## Abstract

A transdermal patch preparation of the β_2_ agonist tulobuterol has been designed to yield sustained β_2_ agonistic effects and has been used as a long-acting β_2_ agonist (LABA) in Japan. LABAs reduce the frequency of exacerbations of chronic obstructive pulmonary disease and bronchial asthma. However, inhibitory effects of LABAs on the replication of rhinovirus (RV), the major cause of exacerbations, have not been demonstrated. To examine the effects of tulobuterol on RV replication and on the production of the replication-induced pro-inflammatory cytokines, human tracheal epithelial cells were infected with a major group RV, type 14 rhinovirus (RV14). Tulobuterol reduced the RV14 titers and RNA levels; the concentrations of cytokines, including interleukin (IL)-1β, IL-6, and IL-8, in the supernatants; and susceptibility to RV14 infection. Tulobuterol reduced the expression of intercellular adhesion molecule-1 (ICAM-1), the receptor for RV14, and the number of acidic endosomes in the cells in which RV14 RNA enters the cytoplasm. Tulobuterol inhibited the activation of nuclear factor kappa B (NF-κB) proteins in nuclear extracts. A selective β_2_-adrenergic receptor antagonist, ICI 118551 [erythro-dl-1-(7-methylindan-4-yloxy)-3-isopropylaminobutan-2-ol], reversed the inhibitory effects of tulobuterol on the RV14 titers and RNA levels, the susceptibility to RV14 infection, cytokine production, and ICAM-1 expression. Tulobuterol may inhibit RV replication by reducing ICAM-1 expression and acidic endosomes and modulate airway inflammation during RV replication.

## Introduction

Rhinoviruses (RVs) are the major cause of the common cold as well as the most common acute infection illnesses in humans (Turner and Couch 2006). RVs are also associated with exacerbations of inflammatory chronic pulmonary diseases such as chronic obstructive pulmonary disease (COPD) (Seemungal et al. 2000) and bronchial asthma (Johnston et al. 1995). Several mechanisms of RV-induced exacerbations of these diseases have been proposed, including virus-induced mucus hypersecretion, airway inflammation (Pizzichini et al. 1998; Seemungal et al. 2000), mast cell activation, and smooth muscle contraction.

Short-acting and long-acting β_2_ agonists (LABAs) improve the symptoms and lung function in patients with bronchial asthma and COPD. Furthermore, LABAs by themselves, or in combination with inhaled corticosteroids reduce the frequency of exacerbations in patients with COPD (Calverley et al. 2007) and bronchial asthma (Pauwels et al. 1997). It has been suggested that these clinical benefits of β_2_ agonists are related to the various effects of the agents, including bronchodilation and antiinflammatory effects (Johnson 1991), improvement of mucociliary clearance and mucosal edema, and inhibition of mucus hypersecretion (Rogers and Barnes 2006).

RV infection induces the production of cytokines and monokines including interleukin (IL)-1, IL-6, and IL-8 (Subauste et al. 1995; Zhu et al. 1996). These cytokines and monokines have pro-inflammatory effects (Akira et al. 1990) and may also be involved in the pathogenesis of RV infections and the infection-induced exacerbations of bronchial asthma and COPD. However, Edwards et al. (2006, 2007) demonstrated that a LABA, salmeterol, increased IL-6 production, had no effects on IL-8 production in a bronchial epithelial cell line (BEAS-2B) cells and primary cultures of normal bronchial epithelial cells, and increased CXCL5 expression in primary cells after RV infection. In contrast, in primary cultures of normal bronchial epithelial cells, salmeterol alone reduced the RV-induced production of RANTES (regulated on activation, normal T cells expressed and secreted/CCL5) and interferon-γ-inducible protein 10 (IP-10/CXCL10). Skevaki et al. (2009) demonstrated that the LABA formoterol reduced the release of IL-8 but had no effect on the release of IL-6 from BEAS-2B cells. Thus, the effects of LABAs on the RV infection-induced production of pro-inflammatory cytokines are still controversial.

The major group RVs enter the cytoplasm of infected cells after binding to receptor intercellular adhesion molecule (ICAM)-1 (Greve et al. 1989; Casasnovas and Springer 1994). The entry of the RNA from this group into the cytoplasm of infected cells is suggested to be mediated by destabilization from receptor binding and endosomal acidification (Casasnovas and Springer 1994). Several agents, including glucocorticoids (Suzuki et al. 2000), macrolide antibiotics, bafilomycin (Pérez and Carrasco 1993), and erythromycin (Suzuki et al. 2002), inhibit the replication of the major group RVs through the reduction of ICAM-1 expression or by increasing the endosomal pH. We have demonstrated that the short-acting β_2_ agonist procaterol inhibits RV replication in human tracheal epithelial cells (Yamaya et al. 2011). However, inhibitory effects of LABAs on RV replication have not been demonstrated.

The tulobuterol patch is a transdermal patch preparation of the β_2_ agonist tulobuterol. The patch is designed to yield sustained β_2_ agonistic effects for 24 hours when applied once daily. The tulobuterol patch has been used as a LABA in Japan and has been reported to improve the quality of life in COPD patients (Fukuchi et al. 2005). In the present study, we examined the effects of tulobuterol on the replication of a major group RV, RV14, in primary cultures of human tracheal epithelial cells. We also examined the effects of tulobuterol on the production of ICAM-1 and on the endosomal pH to clarify the mechanisms responsible for the inhibition of RV14 replication.

## Materials and Methods

### Human tracheal epithelial cell culture

Human tracheal surface epithelial cells were isolated and cultured as described previously (Yamaya et al. 2011). To enhance RV14 release from the cells and to clarify the inhibitory effects of tulobuterol on viral release, the cells were cultured in rolling tubes (Turner and Couch 2006; Yamaya et al. 2011, 2012). To study the effects of tulobuterol on NF-kappa B (NF-κB) activation and acidic endosomes before RV infection, the cells were cultured under stationary conditions.

Trachea samples for cell cultures were obtained after death from 41 patients (age, 73 ± 3 years; 15 females, 26 males). No patients had bronchial asthma, but three patients had COPD. The causes of death were malignant tumors other than lung cancer (*n* = 23), acute myocardial infarction (*n* = 6), congestive heart failure (*n* = 4), renal failure (*n* = 3), cerebral bleeding (*n* = 2), dermatomyositis (*n* = 2), and cerebral infarction (*n* = 1). Of the 41 patients, 15 were ex-smokers and 26 had never smoked. This study was approved by the Tohoku University Ethics Committee.

### Culturing human embryonic fibroblast cells

Human embryonic fibroblast cells (HFL-III cells, Riken Bio Resource Center Cell Bank, Cell No: RCB0523; Tsukuba, Japan) were cultured as described previously (Yamaya et al. 2011).

### Viral stocks

RV14 stocks were prepared from a patient with a common cold by infecting human embryonic fibroblast cells as previously described (Numazaki et al. 1987).

### Detection and titration of viruses

RV14 in supernatant fluids (supernatants) was detected and titrated using the endpoint method (Condit 2006) as previously described (Yamaya et al. 2011). TCID_50_ (tissue culture infective dose) was calculated as previously described (Condit 2006). The rates of change in RV14 concentration in the supernatants are expressed as TCID_50_ units/ml/24 h (Yamaya et al. 2011).

### Quantification of RV RNA

To quantify the RV14 RNA and ribosomal RNA (18S, rRNA) expression in the human tracheal epithelial cells after RV14 infection, two-step real-time quantitative reverse transcription-polymerase chain reaction (RT)-PCR using the Taqman technique (Roche Molecular Diagnostic Systems, Alameda, CA) was performed with TaqMan® Gene Expression Master Mix (Applied Biosystems, Bedford, CA) (Yamaya et al. 2011) according to methods previously described by Nolan et al. (2006).

### Viral infection of the epithelial cells

Infection of the human tracheal epithelial cells with a stock solution of RV14 (100 μL in each tube, 1.0 × 10^4^ TCID_50_ units/100 μL, 5.0 × 10^−2^ TCID_50_ units/cell) was performed as previously described (Yamaya et al. 2011). The cells were infected with RV14 at 3 days (72 h) after treatment with tulobuterol (0.1 μmol/L), except where we describe other concentrations or treatment periods.

### Treatment with tulobuterol

To examine the effects of tulobuterol, cultured human tracheal epithelial cells from the same donors were treated with either tulobuterol (0.1 μmol/L, supplied from Abbott-Japan Co., Ltd., Tokyo, Japan) or the vehicle (0.001% ethanol) from 3 days (72 h) before RV14 infection until the end of the experiments after RV14 infection (Yamaya et al. 2011), except where we describe other concentrations or treatment periods. This particular dose used was chosen because Ruff et al. (1988) demonstrated smooth muscle relaxation of guinea pig tracheae at 0.1 μmol/L or greater.

To examine the concentration-dependent effects of tulobuterol on RV14 replication and acidic endosomes, the cells were treated with tulobuterol at concentrations ranging from 1 nmol/L to 10 μmol/L. Similarly, to examine the time-dependent effects of tulobuterol on RV14 replication and acidic endosomes, the cells were treated with tulobuterol (0.1 μmol/L) for time periods ranging from 0 to 3 days (72 h).

To examine the effects of tulobuterol on ICAM-1 mRNA expression in the cells and the concentration of a soluble form of ICAM-1 (sICAM-1) in supernatants, cells were pretreated with tulobuterol (0.1 μmol/L) for 3 days before RV14 infection, the supernatants were collected, and RNA was extracted from a sample of the cells just prior to infections.

### Collection of supernatants for measurements

The time course of viral release was measured using previously described methods (Yamaya et al. 2011). The supernatants were initially collected at 1 day (24 h) after infection, and then fresh medium with or without tulobuterol was added. Supernatants were also collected at 3 days (72 h) and 5 days (120 h) after infection, and fresh medium with or without tulobuterol was added. Similarly, the supernatants were collected at 7 days (168 h).

Furthermore, to measure RV14 release during the first 24 h, the supernatants were collected at 1 h after RV14 infection, and fresh medium with or without tulobuterol was added. The supernatants were also collected at either 12 h or 24 h after RV14 infection, and fresh medium with or without tulobuterol was added.

Similarly, to examine the effects of tulobuterol on the secretion of IL-1β, IL-6, and IL-8, supernatants were collected just before infection, and 1 day (24 h), 3 days (72 h), and 5 days (120 h) after RV14 infection.

### Effects of tulobuterol on susceptibility to RV infection

The effects of tulobuterol (0.1 μmol/L) on the susceptibility to RV14 infection were evaluated as previously described (Yamaya et al. 2011).

### Measurement of ICAM-1 expression

The mRNA of ICAM-1 was examined using two-step real-time RT-PCR analysis with the methods described above (*Quantification of rhinovirus RNA*). The concentrations of the soluble form of ICAM-1 (sICAM-1) in supernatants were measured with an enzyme immunoassay (EIA) (Yamaya et al. 2011).

### Measurement of changes in acidic endosomes

The distribution and the fluorescence intensity of acidic endosomes in the cells were measured as previously described, using LysoSensor DND-189 dye (Molecular Probes, Eugene, OR) (Gu et al. 1997; Yamaya et al. 2011). The cells on coverslips in Petri dishes were observed with a fluorescence microscope (OLYMPUS IX70; OLYMPUS Co. Ltd., Tokyo, Japan). The excitation wavelength was 443 nm, and the emitted light from the cells was detected through a 505-nm filter. The fluorescence intensity was calculated using a fluorescence image analyzer system (Lumina Vision®; Mitani Co. Ltd., Fukui, Japan) equipped with a fluorescence microscope. The fluorescence intensity of acidic endosomes was measured in 100 human tracheal epithelial cells, and the mean value of fluorescence intensity was expressed as a percentage of the control value compared with the fluorescence intensity of the cells before any treatment.

We studied the effects of a long treatment period with tulobuterol (0.1 μmol/L, 72 h) on acidic endosomes because the cells were pretreated with tulobuterol for 3 days before RV14 infection, except when we examined the time- or dose-dependent effects or the effects at other concentrations.

### Measurement of cytokine production

We measured IL-1β, IL-6, and IL-8 levels in the supernatants using specific enzyme-linked immunosorbent assays (ELISAs) as previously described (Yamaya et al. 2011) at all time points.

### NF-κB assay

Nuclear extracts from the cells were prepared using a TransFactor Extraction Kit (BD Bioscience/CLONTECH, Mountain View, CA). The presence of the translocated p50, p65, and c-Rel subunits was assayed using a TransFactor Family Colorimetric Kit-NFκB (BD Bioscience/CLONTECH) (Fiorucci et al. 2002; Yamaya et al. 2011).

### Statistical analysis

The results are expressed as the mean ± SE. Statistical analysis was performed using one-way analysis of variance (ANOVA). Subsequent post hoc analysis was performed using Bonferroni's method. For all analyses, values of *P* < 0.05 were considered to be significant. The number of donors (tracheae) from which cultured epithelial cells were used is referred to as *n*.

## Results

### Effects of tulobuterol on RV replication in human tracheal epithelial cells

**Figure 1:**
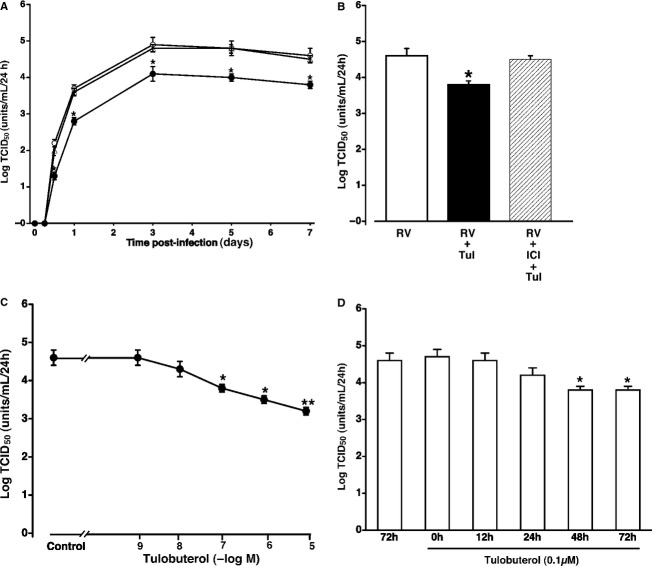
(A) The time course of viral release in the supernatants of human tracheal epithelial cells obtained at different times after exposure to RV14 in the presence of tulobuterol (0.1 μmol/L) (closed circles), tulobuterol (0.1 μmol/L) plus ICI11851 (1 μmol/L) (open triangles), or vehicle (0.001% ethanol) (open circles). The rates of change in RV14 concentration in the supernatants are expressed as TCID_50_ units/mL/24 h. The results are the mean ± SE from six different tracheae (two ex-smokers and four nonsmokers). Significant differences from viral infection alone are indicated by *P < 0.05. (B) Viral release in the supernatants collected over 1 day (24 h) to 3 days (72 h) after infection in the presence of tulobuterol (0.1 μmol/L) (RV + Tul), tulobuterol (0.1 μmol/L) plus ICI11851 (1 μmol/L) (RV + ICI + Tul), or vehicle (RV). The results are the mean ± SE from six different tracheae. Significant differences from viral infection alone are indicated by *P < 0.05. (C) The concentration-response effects of tulobuterol on the viral release in the supernatants collected over 1–3 days after infection in the cells treated with either tulobuterol or vehicle (Control). The results are the mean ± SE from six different tracheae. Significant differences from viral infection alone are indicated by *P < 0.05 and **P < 0.01. (D) Time course of the effects of tulobuterol (0.1 μmol/L) on viral release in the supernatants collected over 1–3 days after infection in the cells treated for times ranging from 0 (0 h) to 3 days (72 h) and the viral release in the cells treated with vehicle for 3 days (72 h). The results are the mean ± SE from five different tracheae (two ex-smokers and three nonsmokers). Significant differences from before any treatment (time 0) are indicated by *P < 0.05.

Exposing confluent human tracheal epithelial cell monolayers to RV14 (5.0 × 10^−2^ TCID_50_ units/cell) consistently led to infection. No virus was detected at 1 h after infection, but RV14 was detected in supernatants at 12 h, and the viral content progressively increased between 1 and 12 h after infection (Fig. 1A). Evidence of continuous viral replication was obtained by demonstrating that each of the supernatants collected at either 12 h to 24 h (1 day), 1 day (24 h) to 3 days (72 h), 3 days (72 h) to 5 days (120 h), or 5 days (120 h) to 7 days (168 h) after infection contained significant levels of RV14 (Fig. 1A). The viral titer levels in the supernatants increased significantly with time for the first 3 days (72 h) (*P* < 0.05 by ANOVA). Furthermore, in the tracheal cells from subjects whose cells were infected with RV14, the supernatants collected during 1 (24 h) to 3 days (72 h) after infection contained consistent levels of RV14 (4.52 ± 0.24 log TCID_50_ units/ml/24 h, *n* = 38).

Treatment of the cells with tulobuterol (0.1 μmol/L) significantly decreased the viral titers of RV14 in the supernatants from 12 h after infection compared with the titers in the cells treated with vehicle (0.001% ethanol) (Fig. 1A and B).

Furthermore, the selective β_2_-adrenergic receptor antagonist ICI 118551 (1 μmol/L, Sigma, St. Louis, MO) (Suzuki et al. 2001) reversed the inhibitory effects of tulobuterol (0.1 μmol/L) on the RV14 titer levels (Fig. 1A and B), whereas ICI 11851 alone did not alter the titer levels (data not shown).

Tulobuterol reduced RV14 release in a concentration-dependent manner (Fig. 1C). Pretreatment of the cells with tulobuterol reduced the viral titers of RV14 in the supernatants at concentrations of 0.1 μmol/L or greater (Fig. 1C).

The inhibitory effects of tulobuterol on RV14 release were time-dependent. The maximum inhibitory effect was obtained when the cells were pretreated with tulobuterol for 3 days (72 h) (Fig. 1D). Significant inhibitory effects on RV14 release were observed when the cells were treated with tulobuterol (0.1 μmol/L) for 48 h or longer prior to RV14 infection (Fig. 1D).

The RV14 titer levels in the supernatants of cells collected from 13 ex-smokers over 1 day (24 h) to 3 days (72 h) after infection did not differ from those of the 25 patients who had never smoked (4.58 ± 0.32 log TCID_50_ units/ml/24 h vs. 4.50 ± 0.21 log TCID_50_ units/ml/24 h, respectively, *P* > 0.02). Likewise, the RV14 titer levels in the supernatants of the cells from the three patients who had COPD did not differ from those of the 35 patients without COPD (data not shown). No virus was detected in the supernatants after infection with ultraviolet (UV)-inactivated RV14 (data not shown).

Treatment with tulobuterol (0.1 μmol/L) for 3 days (72 h) did not change viability (99 ± 1% in tulobuterol vs. 99 ± 1% in vehicle, *n* = 5, *P* > 0.50), as assessed by trypan blue exclusion. Furthermore, until 7 days (168 h) after initiation of cell culture, the cells made confluent sheets in the tubes in both the culture medium alone and the medium containing tulobuterol (0.1 μmol/L) at the same time points. The number of cells in the confluent sheets cultured in the medium supplemented with tulobuterol (0.1 μmol/L) did not differ from that in the medium supplemented with vehicle (2.1 ± 0.3 × 10^6^ of cells/tube in tulobuterol vs. 2.2 ± 0.3 × 10^6^ of cells/tube in vehicle, *n* = 5, *P* > 0.50). When lactate dehydrogenase (LDH) concentrations in the supernatants 3 days (72 h) after tulobuterol treatment were measured, the treatment with tulobuterol (0.1 μmol/L) for 3 days (72 h) did not appear to alter the LDH concentration (29 ± 2 IU/mL/24 h in tulobuterol vs. 30 ± 2 IU/mL/24 h in vehicle, *n* = 5, *P* > 0.50).

### Effects of tulobuterol on viral RNA as measured by real-time RT-PCR

**Figure 2:**
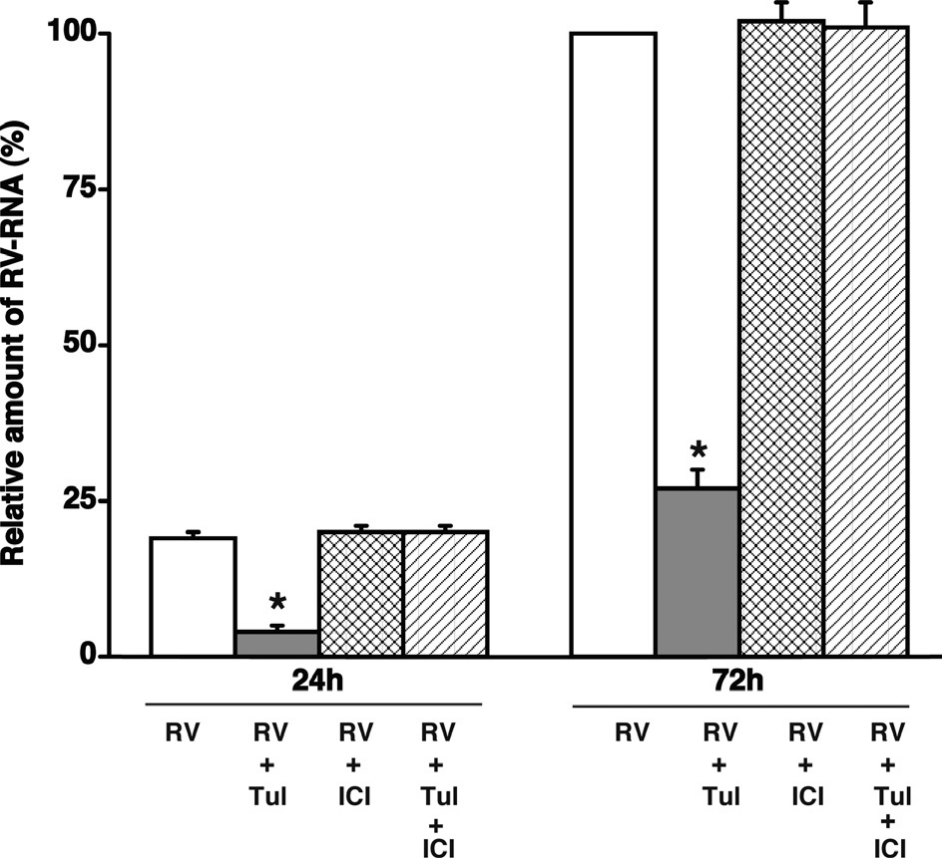
Replication of viral RNA in human tracheal epithelial cells at 1 day or 3 days after infection with RV14 in the presence of tulobuterol (0.1 μmol/L) (RV + Tul), ICI 118551 (1 μmol/L) (RV + ICI), tulobuterol plus ICI 118551 (RV + Tul + ICI), or vehicle (RV) as detected by real-time quantitative RT-PCR. The results are expressed as the relative amount of RNA expression (%) compared with that of maximal RV14 RNA at day 3 in the cells treated with vehicle and are reported as the mean ± SE from five samples (two ex-smokers and three nonsmokers). Significant differences from treatment with vehicle (RV) at each time are indicated by *P < 0.05.

Further evidence of the inhibitory effects of tulobuterol on RV14 RNA replication in human tracheal epithelial cells was provided by real-time quantitative RT-PCR analysis. RNA extraction was performed at 1 day (24 h) and 3 days (72 h) after RV14 infection. RV14 RNA in the cells was consistently observed from 1 day (24 h) after infection and increased between 1 day (24 h) and 3 days (72 h) after infection (Fig. 2). The maximum level of RV14 RNA replication was observed at 3 days (72 h) after infection, whereas RV14 RNA in the cells was not observed before infection. Tulobuterol (0.1 μmol/L) decreased the RV14 RNA levels at 1 day (24 h) and at 3 days (72 h) after infection (Fig. 2).

However, ICI 118551 (1 μmol/L) reversed the inhibitory effects of tulobuterol (0.1 μM) on RV14 RNA replication, whereas ICI 118551 alone did not alter RNA replication (Fig. 2). The levels of RV14 RNA in the cells treated with ICI 118551 (1 μmol/L) alone did not differ from the levels in the cells treated with vehicle (0.001% ethanol) at 1 day (24 h) and 3 days (72 h) after RV14 infection (Fig. 2). In contrast, the level of RV14 RNA in the cells treated with tulobuterol (0.1 μmol/L) plus ICI 118551 (1 μmol/L) was significantly higher than the level in the cells treated with tulobuterol alone and did not differ from the level in the cells treated with vehicle at 1 day (24 h) and at 3 days (72 h) after RV14 infection (Fig. 2).

### Effects of tulobuterol on susceptibility to RV infection

Treatment of the cells with tulobuterol (0.1 μmol/L) decreased their susceptibility to RV14 infection. When viral release was measured using supernatants collected 3 days (72 h) after RV14 infection, the minimum dose of RV14 necessary to cause infection in the cells treated with tulobuterol (0.1 μmol/L, 72 h) (3.3 ± 0.2 log TCID_50_ units/mL, *n* = 5, *P* < 0.05) was significantly higher than the minimum dose in the cells treated with vehicle (0.001% ethanol) (2.4 ± 0.2 log TCID_50_ units/mL, *n* = 5).

The selective β_2_-adrenergic receptor antagonist ICI 118551 (1 μmol/L) by itself did not alter the minimum dose of RV14 necessary to cause viral release in the supernatants of the cells and did not affect susceptibility (data not shown). In contrast, ICI 118551 reversed the effects of tulobuterol on susceptibility to RV14 infection. The treatment of the cells with tulobuterol (0.1 μmol/L) plus ICI 118551 (1 μmol/L) decreased the minimum dose of RV14 necessary to cause viral release in the supernatants of the cells (2.5 ± 0.2 log TCID_50_ units/mL, *n* = 5) compared with the dose in the cells treated with tulobuterol (*P* < 0.05) to the levels in the cells treated with the tulobuterol vehicle (0.001% ethanol).

### Effects of tulobuterol on the expression of ICAM-1

**Figure 3:**
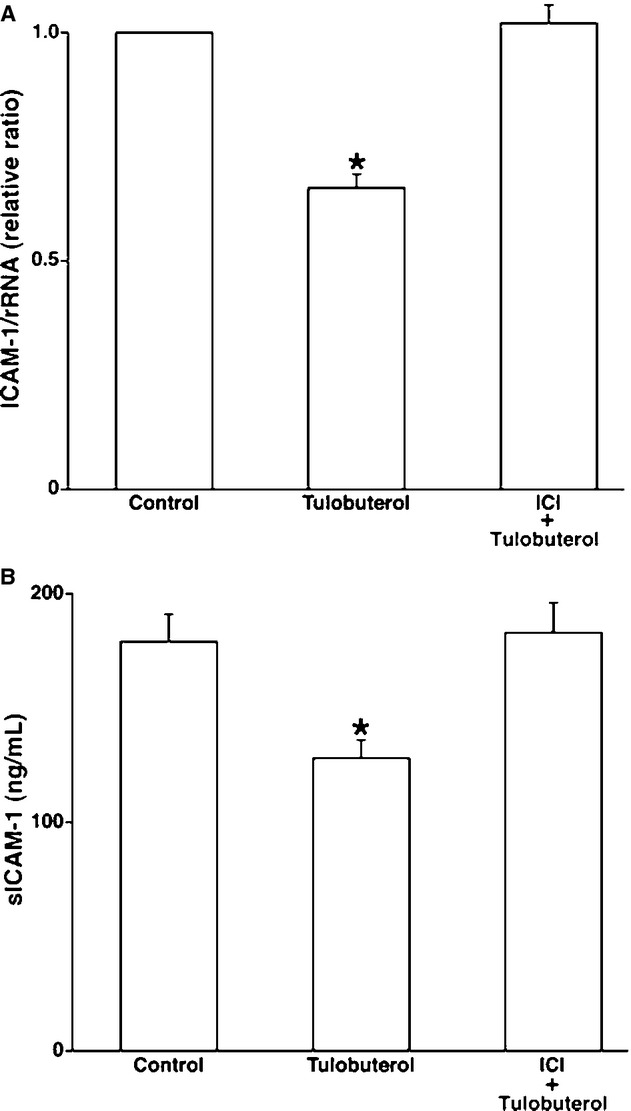
(A) The expression of ICAM-1 mRNA before RV14 infection in human tracheal epithelial cells treated with tulobuterol (0.1 μmol/L) (Tulobuterol), tulobuterol plus ICI 118551 (1 μmol/L) (ICI + Tul), or the vehicle of tulobuterol (Control) for 3 days. The ICAM-1 mRNA was normalized to the constitutive expression of ribosomal RNA (rRNA). The expression of ICAM-1 mRNA in the cells treated with vehicle (Control) was set to 1.0. The results are the mean ± SE from five different tracheae (two ex-smokers and three nonsmokers). Significant differences from control values are indicated by *P < 0.05. (B) The sICAM-1 concentrations in the supernatants before RV14 infection in human tracheal epithelial cells treated with tulobuterol (0.1 μmol) (Tul), tulobuterol plus ICI 118,551 (1 μmol/L) (ICI + Tul), or vehicle of tulobuterol (Control) for 3 days. The concentrations of sICAM-1 in the supernatants are expressed as ng/mL. The results are the mean ± SE from five different tracheae. Significant differences from control values are indicated by *P < 0.05.

Tulobuterol (0.1 μmol/L, 72 h) reduced the baseline ICAM-1 mRNA expression in the cells by approximately 35% compared with that of the cells treated with the tulobuterol vehicle (0.001% ethanol) before RV14 infection (Fig. 3A). Furthermore, the concentrations of sICAM-1 in the supernatants of the cells treated with tulobuterol (0.1 μmol/L) were significantly lower than those in the cells treated with vehicle before RV14 infection (Fig. 3B).

ICI 118551 (1 μmol/L) itself did not change ICAM-1 mRNA expression and sICAM-1 release in the supernatants (data not shown). In contrast, ICI 118551 (1 μmol/L) reversed the inhibitory effects of tulobuterol on the ICAM-1 mRNA expression in the cells and sICAM-1 release in the supernatants (Fig. 3A and B). The ICAM-1 mRNA expression level and the concentration of sICAM-1 in the supernatants of the cells treated with tulobuterol (0.1 μmol/L) plus ICI 118551 (1 μmol/L) were significantly higher than those in the cells treated with tulobuterol (0.1 μmol/L) alone and did not differ from the expression level and concentration in the cells treated with vehicle before RV14 infection (Fig. 3A and B).

### Effects of tulobuterol on the acidification of endosomes

**Figure 4:**
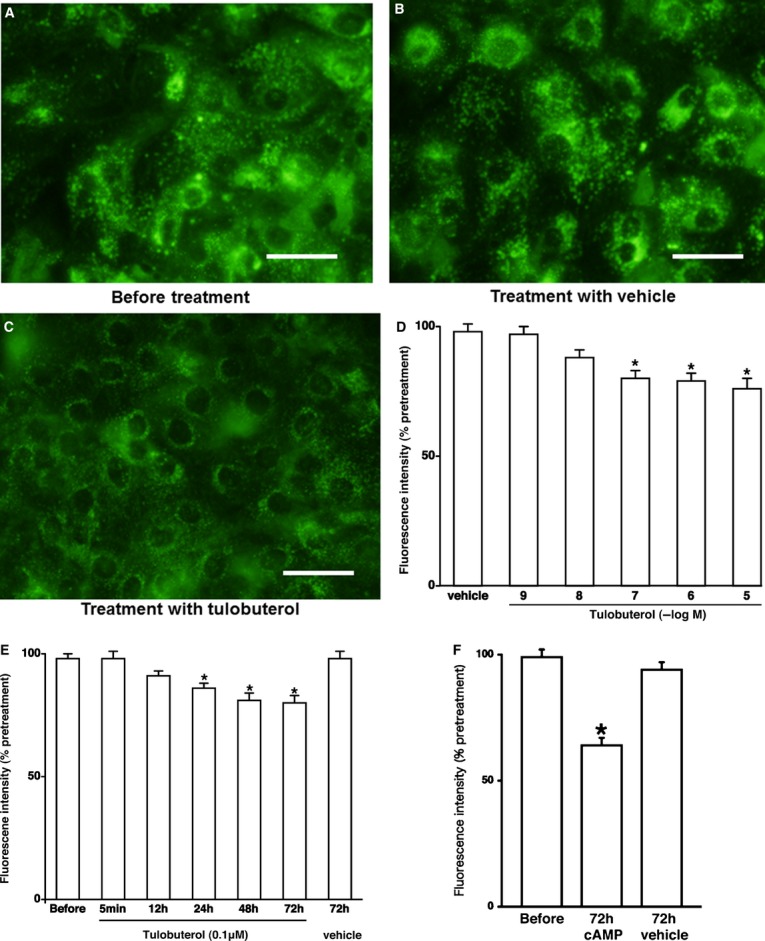
(A–C) Changes in the distribution of acidic endosomes with green fluorescence in human tracheal epithelial cells before (A) and 3 days (72 h) after treatment with tulobuterol (0.1 μmol/L) (C) or vehicle (B). The data are representative of five different experiments (two ex-smokers and three nonsmokers). (Bar = 100 μm) (D) The concentration-response effects of tulobuterol on the fluorescence intensity of acidic endosomes 3 days after treatment. The results are the mean ± SE from five different tracheae. Significant differences from vehicle alone (Vehicle) are indicated by *P < 0.05. (E) The time course of the effects of tulobuterol (0.1 μmol/L) on the fluorescence intensity of acidic endosomes in the cells treated for times ranging from 0 (Before) to 3 days (72 h) and the fluorescence intensity in the cells treated with vehicle of tulobuterol (vehicle) for 3 days. The results are the mean ± SE from five different tracheae. Significant differences from before any treatment (Before) are indicated by *P < 0.05. (F) The effects of dibutyryl cAMP (100 μmol/L) (cAMP) on the fluorescence intensity of acidic endosomes 3 days after treatment. The results are the mean ± SE from three different tracheae (one ex-smoker and two nonsmokers). Significant differences from before any treatment (Before) are indicated by *P < 0.05.

Acidic endosomes in human tracheal epithelial cells were stained green with LysoSensor DND-189 (Fig. 4A–C) as described previously (Yamaya et al. 2011). Treatment with vehicle (0.001% ethanol) for 3 days (72 h) did not change the number of acidic endosomes presenting green fluorescence in the cells (Fig. 4A and B) or the fluorescence intensity of acidic endosomes (Fig. 4D and E) compared with the intensity in the cells before any treatment. In contrast, treatment with tulobuterol (0.1 μmol/L, 72 h) reduced the number of acidic endosomes with green fluorescence in the cells (Fig. 4C) and the fluorescence intensity of acidic endosomes in the cells (Fig. 4D and E) compared with cells treated with vehicle and before any treatment.

The inhibitory effects of tulobuterol on the fluorescence intensity of acidic endosomes were dose dependent. Significant inhibitory effects were observed at 0.1 μmol/L or greater, and the maximum inhibitory effect was obtained at 10 μmol/L (Fig. 4D). The inhibitory effects of tulobuterol on the fluorescence intensity of acidic endosomes were also time dependent, and significant inhibitory effects were observed when cells were treated with tulobuterol (0.1 μmol/L) for 24 h or longer (Fig. 4E). The maximum inhibitory effect was obtained when the cells were treated with tulobuterol for 3 days (72 h) (Fig. 4E).

We also examined the effects of dibutyryl cyclic-AMP (dibutyryl cAMP, Sigma) on the acidic endosomes to examine the mechanisms of tulobuterol-induced increases in endosomal pH. Treatment with dibutyryl cAMP (100 μmol/L) (Gekle et al. 2002) for 72 h reduced the number of acidic endosomes (data not shown) and the fluorescence intensity of acidic endosomes (Fig. 4F). The fluorescence intensity in the cells treated with vehicle did not differ from the intensity observed before any treatment (Fig. 4F).

### Effects of tulobuterol on cytokine production

**Figure 5:**
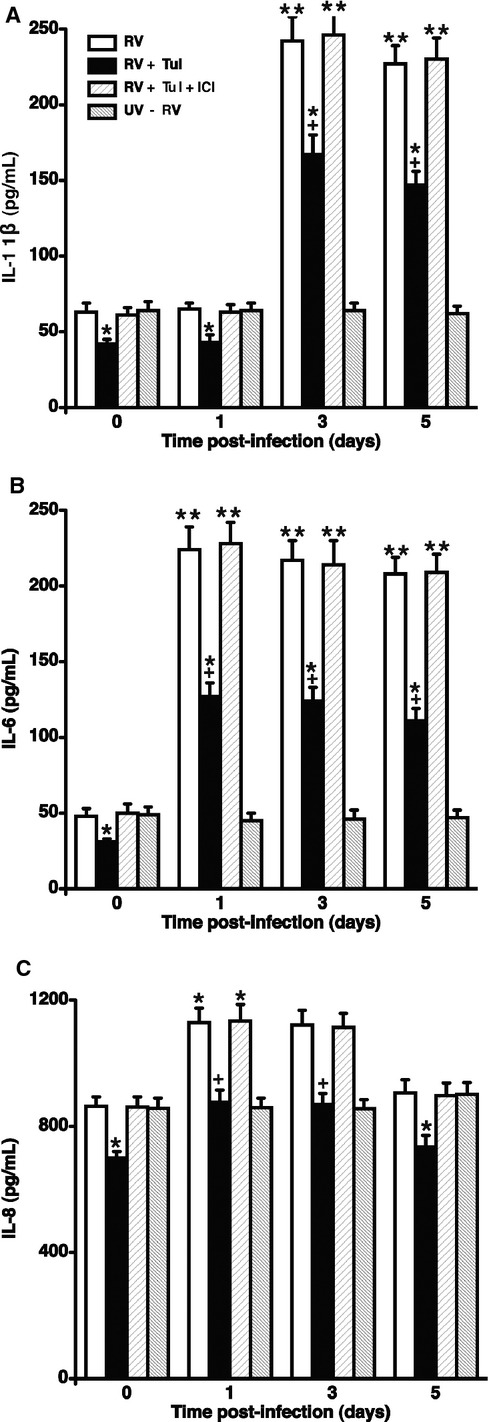
(A–C) Time course changes in the release of cytokines into the supernatants of human tracheal epithelial cells before and after RV14 infection in the presence of tulobuterol (0.1 μmol/L) (RV + Tul), tulobuterol plus ICI 118551 (1 μmol/L) (RV + Tul + ICI), or vehicle (RV). The results are the mean ± SE from six different tracheae (three ex-smokers and three nonsmokers). Significant differences from values before RV14 infection (time 0) in the presence of vehicle are indicated by *P < 0.05 and **P < 0.01. Significant differences from RV14 infection alone (RV) at each time point after infection are indicated by +*P* < 0.05.

Tulobuterol (0.1 μmol/L) reduced the baseline secretion of IL-1β, IL-6, and IL-8 for 24 h before RV14 infection compared with the levels observed in cells treated with vehicle (0.001% ethanol) (Fig. 5). RV14 infection increased the secretion of IL-1β, IL-6, and IL-8. Maximum secretion was observed at 1 day (24 h) after RV14 infection for IL-6 and IL-8 and at 3 days (72 h) after infection for IL-1β. Tulobuterol (0.1 μmol/L) also reduced the RV14 infection-induced secretion of IL-1β, IL-6, and IL-8 compared with the levels observed in the cells treated with vehicle (Fig. 5). UV-inactivated RV14 infection did not affect the secretion of these cytokines.

Treatment of the cells with ICI 118551 (1 μmol/L, 72 h) reversed the inhibitory effects of tulobuterol on the baseline and RV14 infection-induced secretion of IL-1β, IL-6, and IL-8 (Fig. 5), whereas ICI 118551 alone did not affect the secretion of these cytokines (data not shown).

In contrast, ultraviolet-irradiated RV14 did not increase the expression of IL-1β, IL-6, and IL-8 at any time point after infection (Fig. 5). The secretion of IL-1β, IL-6, and IL-8 in the supernatants of cells from three ex-smokers did not differ from those of cells from three patients who had never smoked (data not shown). Similarly, the secretion of IL-1β, IL-6, and IL-8 in the supernatants of cells from three patients who had COPD did not differ from those from three ex-smokers without COPD complications (data not shown).

### Effects on NF-κB

**Figure 6:**
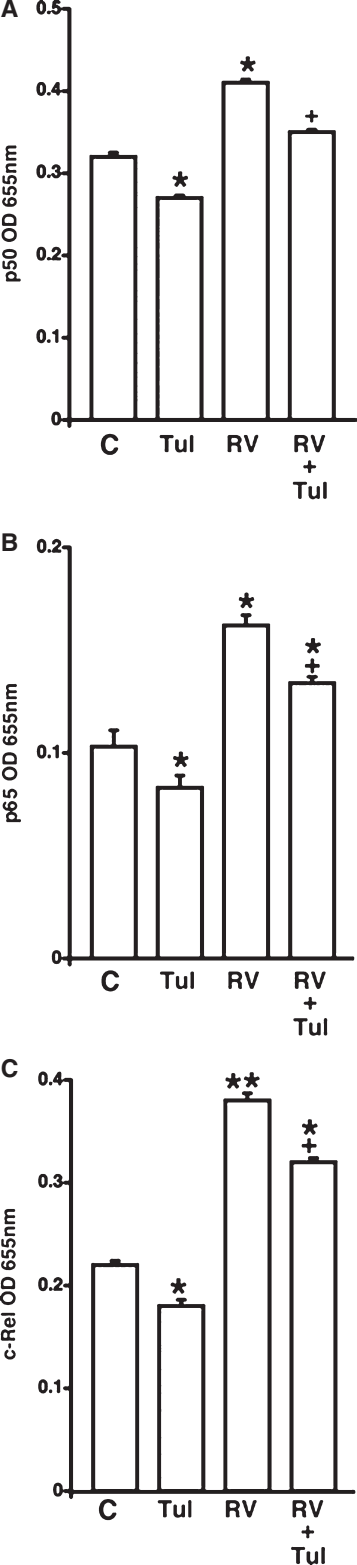
(A–C) Amount of p50 (A), p65 (B), and c-Rel (C) in nuclear extracts in human tracheal epithelial cells treated with tulobuterol (1.0 μmol/L) (Tul) or vehicle (C) for 3 days (72 h) before RV14 infection, and the amount in the cells 3 days after infection with RV14 in the presence of tulobuterol (RV + Tul) or in the presence of vehicle (RV). The results are expressed as the OD and represent the mean ± SE from four different tracheae (two ex-smokers and two nonsmokers). Significant differences from control values (C) before RV14 infection are indicated by *P < 0.05 and **P < 0.05. Significant differences from RV infection alone (RV) are indicated by +*P* < 0.05.

In cultured human tracheal epithelial cells, tulobuterol (1.0 μmol/L, 72 h) produced a small but significant reduction of the amount of p50, p65, and c-Rel of NF-κB in the nuclear extracts compared with the levels observed in the cells treated with vehicle (Fig. 6A–C) and observed in the cells prior to RV14 infection (data not shown). RV14 infection increased the amount of p50, p65, and c-Rel of NF-κB in the nuclear extracts in the cells (Fig. 6A–C). Likewise, tulobuterol (1.0 μmol/L, 72 h) treatment produced a small but significant reduction in the amount of p50, p65, and c-Rel of NF-κB induced by RV14 infection (Fig. 6A–C).

## Discussion

In this study, we demonstrated that tulobuterol reduced the titers of a major group RV, RV14, in supernatants and also reduced RNA replication of the virus in primary cultures of human tracheal epithelial cells. Pretreatment with tulobuterol reduced the expression of ICAM-1, the receptor for the major group RVs (Greve et al. 1989), and increased the minimum dose of RV14 necessary to cause viral infection. A selective β_2_-adrenergic receptor antagonist, ICI 118551 (Yamaya et al. 2011), reversed the inhibitory effects of tulobuterol on RV14 titer levels, viral RNA replication, and the expression of ICAM-1. Treatment with ICI 118551 also reversed the inhibitory effects of tulobuterol on the susceptibility of the cells to RV14 infection. These findings suggest that the β_2_-adrenoceptor-mediated effects of tulobuterol might inhibit RV14 infection partly through reducing the production of its receptor, as previously reported for the inhibitory effects of agents such as dexamethasone and the short-acting β_2_ agonist procaterol (Suzuki et al. 2000; Yamaya et al. 2011).

Tulobuterol reduced the fluorescence intensity of acidic endosomes by 20% and the potency of the inhibitory effects was smaller than that observed with a proton ATPase inhibitor, bafilomycin (Suzuki et al. 2001), and procaterol (Yamaya et al. 2011). The results in the present study suggest that the order of the potency of inhibitory effects on viral replication (bafilomycin > procaterol > tulobuterol) is associated with the potency of the effects on the fluorescence intensity (Suzuki et al. 2001; Yamaya et al. 2011). The exact magnitude, how much the inhibitory effects of tulobuterol on the acidic endosomes were associated with reduction of viral replication, could not be determined. However, these findings suggest that the potency of the inhibitory effects of agents on RV replication was associated with the potency of the effects on the acidic endosomes. A lower reduction in the amount of acidic endosomes by tulobuterol might alter rhinoviral replication, and the tulobuterol-mediated reduction of acidic endosomes might also augment the effects on the reduction of RV14 replication in combination with the inhibitory effects on ICAM-1 expression, the receptor for the RV14.

The endosomal pH is regulated by vacuolar H^+^-ATPase (Mellman et al. 1986) and by ion transport across Na^+^/H^+^ exchangers (Marshansky and Vinay 1996; Nass and Rao 1998). Tulobuterol increases cAMP levels in the cells (Morin et al. 2000), and cAMP increases endosomal pH in kidney epithelial cells through the inhibition of a Na^+^/H^+^ exchanger (Gekle et al. 2002). We also observed that cAMP increases endosomal pH in human tracheal epithelial cells. These findings suggest that tulobuterol may have an inhibitory effect on Na^+^/H^+^ exchangers through the increased production of cAMP.

Tulobuterol reduced viral titers to 10% or less and the antiviral activity of tulobuterol is limited. Similarly, the reduction of the release of cytokines by tulobuterol was moderate, and tulobuterol reduced the release of IL-1β and IL-6 by 40% and 50%, respectively. However, Skevaki et al. (2009) reported that a clinically used inhaled corticosteroid, budesonide, with sufficient antiinflammatory effects, reduces IL-6 in the supernatants of bronchial epithelial cells by approximately 70%. Furthermore, Pan et al. (2006) reported that treatment with budesonide (10^−8^ mol/L) reduces the release of IL-8 by 50% in the supernatants of human bronchial epithelial cells (BEAS-2B) in response to the stimulation with IL-1β plus pyocyanin, a toxic factor from the bacteria *Pseudomonas aeruginosa*. In the present study, tulobuterol reduced the release of IL-8 in supernatants to baseline levels. These findings suggest that the potency of the inhibitory effects of tulobuterol on IL-1β, IL-6, and IL-8 release may be sufficient to inhibit airway inflammation. Therefore, tulobuterol may have antiinflammatory effects that are sufficient to drive an inflammatory response.

Neutrophilic and eosinophilic inflammation in the exacerbation of bronchial asthma and COPD by RV infection has also been associated with a variety of mediators including IL-6 and IL-8 (Pizzichini et al. 1998; Seemungal et al. 2000). Tulobuterol inhibits superoxide anion (O^2−^) production by neutrophils and eosinophils (Yasui et al. 2006) and reduces the number of eosinophils in the sputum of asthma patients (Hozawa et al. 2009), although the levels of IL-8 in the induced sputum are not reduced in COPD patients treated with tulobuterol (Kanehara et al. 2008). Reduced pro-inflammatory cytokine concentrations by tulobuterol during RV14 infection in the present study are consistent with previous findings on the inhibitory effects of procaterol on the plasma levels of cytokines, including IL-1β, in rats (Izeboud et al. 2004). Salmeterol also inhibits the production of pro-inflammatory cytokines and monokines such as RANTES in bronchial epithelial cells after RV infection (Edwards et al. 2006). Similar to the inhibitory effects of procaterol (Yamaya et al. 2011), tulobuterol may modulate the airway inflammation induced by RV infection.

Intercellular adhesion molecule-1 also plays a vital role in the recruitment and migration of immune effector cells to sites of local inflammation observed in patients with bronchial asthma and COPD (Riise et al. 1994; Grunberg and Sterk 1999). The inhibitory effects of tulobuterol on ICAM-1 that we demonstrated in this study are consistent with previous reports that β_2_ agonists, such as fenoterol, salmeterol, and procaterol, reduce ICAM-1 expression in airway epithelial cells and fibroblast cells (Oddera et al. 1998; Silvestri et al. 2001; Yoshida et al. 2009; Yamaya et al. 2011) and may also be associated with the inhibitory effects of LABAs, such as formoterol and salmeterol, on the exacerbations of bronchial asthma and COPD (Pauwels et al. 1997; Calverley et al. 2007). However, the antiinflammatory effects of formoterol and salmeterol are still uncertain.

In the present study, tulobuterol reduced the expression of ICAM-1 and pro-inflammatory cytokines. It has been reported that NF-κB increases the expression of the genes for ICAM-1 and various pro-inflammatory cytokines (Zhu et al. 1996; Papi and Johnston 1999). Tulobuterol administration reduced the levels of p50, p65, and c-Rel of NF-κB in human tracheal epithelial cells in the present study, and these inhibitory effects on NF-κB activity are consistent with those of salmeterol and procaterol in lung myofibroblasts (Baouz et al. 2005) and human tracheal epithelial cells (Yamaya et al. 2011). The results for the NF-κB activation using ELISA-based methods, which were used in the present study, were consistent with those studied with Western blot analysis used to measure the cytosolic amount of IkB-α (Yamaya et al. 2011, 2012). These findings suggest that tulobuterol might reduce the expression of ICAM-1 partly through the reduction of NF-κB activation.

Because tulobuterol inhibited NF-κB activation and reduced cytokine concentration in the supernatants before RV14 infection in the present study, the attenuation of the production of inflammatory cytokines might be due to the inhibition of NF-κB activation by tulobuterol. We previously reported that RV14 replication stimulates NF-κB activation (Suzuki et al. 2002). Therefore, the attenuation of RV14 replication observed in the present study might also be associated with the reduction of cytokine production after RV14 infection.

In contrast, Edwards et al. (2007) reported that salmeterol increases IL-6 production and enhances NF-κB pathway activation following RV infection in a bronchial epithelial cell line (BEAS-2B) and in primary cultures of normal bronchial epithelial cells. Furthermore, another report demonstrated that β_2_ agonists do not affect NF-κB-induced activation of the IL-6 gene in airway smooth muscle cells (Kaur et al. 2008). However, the production of IL-6 after RV infection through the activation of NF-κB has been reported in A549 cells (Zhu et al. 1996). Fragaki et al. (2006) demonstrated that salmeterol plus corticosteroids reduced IL-6 release in response to *Staphylococcus aureus* in a transformed human tracheal gland cell line partly through the inhibition of NF-κB. The inhibition of NF-κB and TNF-α-induced IL-6 production by salmeterol has also been reported in lung myofibroblasts (Baouz et al. 2005). We previously reported that reduced production of IL-6 by the β_2_ agonist procaterol is associated with the inhibition of NF-κB (Yamaya et al. 2011). Thus, these findings suggest that the different effects of β_2_ agonists on IL-6 and NF-κB after RV14 infection or after addition of stimulants may be partly associated with differences in cell type and culture conditions.

In the present study, we observed that tulobuterol reduced the production of ICAM-1 and inflammatory cytokines and that a selective β_2_-adrenergic receptor antagonist ICI 118551 reversed the inhibitory effects of tulobuterol, as reported in a previous study using procaterol (Yamaya et al. 2011). Farmer and Pugin (2000) reported that β-adrenergic agonists increase the cytoplasmic concentration of inhibitory kappa B-α (IκB-α), by decreasing its degradation. Tulobuterol and procaterol increase intracellular cAMP (Morin et al. 2000; Yamaya et al. 2011), although we did not examine the effects of tulobuterol on the production of cAMP. It has been reported that cAMP-induced signals inhibit NF-κB activities (Gerlo et al. 2011). These mechanisms may relate to the tulobuterol-induced inhibitory effects on the NF-κB activity observed in the present study.

Ruff et al. (1988) reported that acetylcholine-induced contraction of the tracheal smooth muscle of guinea pigs was relaxed by tulobuterol and 4-hydroxytulobuterol, a metabolite of tulobuterol (Kubo et al. 1980). They also reported that the potency of 4-hydroxytulobuterol was more than 1000-fold higher than that of tulobuterol (Ruff et al. 1988). The maximum serum concentration of tulobuterol was 2.1–2.4 ng/mL (=10 nmol/L), and the amount of urine excretion of 4-hydroxytulobuterol was approximately half that of tulobuterol (data from Abbott Japan Co. Ltd.), suggesting that the levels of 4-hydroxytulobuterol are similar to those of tulobuterol in serum. We demonstrated that tulobuterol reduced the RV14 titers in supernatants at concentrations ranging from 0.1 μmol/L to 10 μmol/L. These findings suggest that tulobuterol may inhibit RV14 replication at clinically available concentrations, although we could not measure the effects of 4-hydroxytulobuterol.

Because tulobuterol alone did not change the cell viability as assessed by the exclusion of trypan blue and LDH concentrations in supernatants, reduced cytokine release might be partly associated with the inhibition of NF-κB activation but not with cell injury.

In summary, this is the first report that the β_2_-agonist tulobuterol, which in patch form has been used as a LABA in Japan, reduces RV14 titers in supernatants, reduces RV RNA replication in cultured human tracheal epithelial cells, and decreases the susceptibility of the cells to RV14 infection. These results may occur partly through the reduced expression of ICAM-1, the receptor for the major group RVs, and a reduction in the number of acidic endosomes from which RV14 RNA enters the cytoplasm. Tulobuterol reduced the baseline and RV replication-induced release of IL-1β, IL-6, and IL-8 in the supernatants. Tulobuterol may inhibit the replication of the major group RVs and modulate the inflammatory responses in the airways after RV replication.

## References

[b1] Akira S, Hirano T, Taga T, Kishimoto T (1990). Biology of multifunctional cytokines: IL 6 and related molecules (IL 1 and TNF). FASEB J.

[b2] Baouz S, Giron-Michel J, Azzarone B, Giuliani M, Cagnoni F, Olsson S (2005). Lung myofibroblasts as targets of salmeterol and fluticasone propionate: inhibition of α-SMA and NF-kappaB. Int. Immunol.

[b3] Calverley PM, Anderson JA, Celli B, Ferguson GT, Jenkins C, Jones PW (2007). **TORCH investigators** Salmeterol and fluticasone propionate and survival in chronic obstructive pulmonary disease. N. Engl. J. Med.

[b4] Casasnovas JM, Springer TA (1994). Pathway of rhinovirus disruption by soluble intercellular adhesion molecule 1 (ICAM-1): an intermediate in which ICAM-1 is bound and RNA is released. J. Virol.

[b5] Condit RC, Knipe DM, Howley PM (2006). Principles of Virology. Fields virology.

[b6] Edwards MR, Johnson MW, Johnston SL (2006). Combination therapy: synergistic suppression of virus-induced chemokines in airway epithelial cells. Am. J. Respir. Cell Mol. Biol.

[b7] Edwards MR, Haas J, Johnson RA, Panettieri M, Johnston SL (2007). Corticosteroids and β_2_ agonists differentially regulate rhinovirus-induced interleukin-6 via distinct cis-acting elements. J. Biol. Chem.

[b8] Farmer P, Pugin J (2000). β-Adrenergic agonists exert their “anti-inflammatory” effects in monocytic cells through the IκB/NF-κB pathway. Am. J. Physiol.

[b9] Fiorucci S, Antonelli E, Distrutti E, Flower P, Del Soldato RJ, Clark MJ (2002). NCX-1015, a nitric-oxide derivative of prednisolone, enhances regulatory T cells in the lamina propria and protects against 2,4,6-trinitrobenzene sulfonic acid-induced colitis in mice. Proc. Natl. Acad. Sci. USA.

[b10] Fragaki K, Kileztky C, Trentesaux C, Zahm JM, Bajolet O, Johnson M (2006). Downregulation by a long-acting β_2_-adrenergic receptor agonist and corticosteroid of *Staphylococcus aureus*-induced airway inflammatory mediator production. Am. J. Physiol.

[b11] Fukuchi Y, Nagai A, Seyama K, Nishimura M, Hirata K, Kubo K (2005). Clinical efficacy and safety of transdermal tulobuterol in the treatment of stable COPD: an open-label comparison with inhaled salmeterol. Treat. Respir. Med.

[b12] Gekle M, Serrano OK, Drumm K, Mildenberger S, Freudinger R, Gassner B (2002). NHE3 serves as a molecular tool for cAMP-mediated regulation of receptor-mediated endocytosis. Am. J. Physiol.

[b13] Gerlo S, Kooijman R, Beck IM, Kolmus K, Spooren A, Haegeman G (2011). Cyclic AMP: a selective modulator of NF-κB action. Cell. Mol. Life Sci.

[b14] Greve JM, Davis G, Meyer AM, Forte CP, Yost SC, Marlor CW (1989). The major human rhinovirus receptor is ICAM-1. Cell.

[b15] Grunberg K, Sterk PJ (1999). Rhinovirus infections: induction and modulation of airways inflammation in asthma. Clin. Exp. Allergy.

[b16] Gu F, Aniento F, Parton RG, Gruenberg J (1997). Functional dissection of COP-I subunits in the biogenesis of multivesicular endosomes. J. Cell Biol.

[b17] Hozawa S, Haruta Y, Terada M, Yamakido M (2009). Effects of addition of beta2-agonist tulobuterol patches to inhaled corticosteroid in patients with asthma. Allergol. Int.

[b18] Izeboud CA, Hoebe KH, Grootendorst AF, Nijmeijer SM, Witkamp AS, van Miert RR (2004). Endotoxin-induced liver damage in rats is minimized by β_2_-adrenoceptor stimulation. Inflamm. Res.

[b19] Johnson M (1991). Salmeterol: a novel drug for the treatment of asthma. Agents Actions (Suppl.).

[b20] Johnston SL, Pattemore PK, Sanderson G, Smith S, Lampe F, Josephs L (1995). Community study of role of viral infections in exacerbations of asthma in 9-11 year old children. Br. Med. J.

[b21] Kanehara M, Yokoyama A, Tomoda Y, Shiota N, Iwamoto H, Ishikawa N (2008). Anti-inflammatory effects and clinical efficacy of theophylline and tulobuterol in mild-to-moderate chronic obstructive pulmonary disease. Pulm. Pharmacol. Ther.

[b22] Kaur M, Holden NS, Wilson SM, Sukkar MB, Chung KF, Barnes PJ (2008). Effects of β_2_-adrenoceptor agonists and other cAMP-elevating agents on inflammatory gene expression in human ASM cells: a role for protein kinase A. Am. J. Physiol.

[b23] Kubo S, Matsubara I, Yamasaki M, Kasamatsu S, Koshinaka E, Kato H (1980). Pharmacological studies of 1-(2-chloro-4-hydroxyphenyl)-t-butylaminoethanol (Hoku-81), a new bronchodilator. Arzneim.-Forsch./ Drug Res.

[b24] Marshansky V, Vinay P (1996). Proton gradient formation in early endosomes from proximal tubes. Biochim. Biophys. Acta.

[b25] Mellman I, Fuchs R, Helenius A (1986). Acidification of the endocytic and exocytic pathways. Ann. Rev. Biochem.

[b26] Morin D, Sapena R, Tillement JP, Urien S (2000). Evidence for different interactions between β_1_- and β_2_-adrenoceptor subtypes with adenylate cyclase in the rat brain: a concentration-response study using forskolin. Pharamacol. Res.

[b27] Nass R, Rao R (1998). Novel localization of a Na^+^/H^+^ exchanger in a late endosomal compartment of yeast. Implications for vacuole biogenesis. J. Biol. Chem.

[b28] Nolan T, Hands RE, Bustin SA (2006). Quantification of mRNA using real-time RT-PCR. Nat. Protoc.

[b29] Numazaki Y, Oshima T, Ohmi A, Tanaka A, Oizumi Y, Komatsu S (1987). A microplate methods for isolation of viruses from infants and children with acute respiratory infections. Microbiol. Immunol.

[b30] Oddera S, Silvestri M, Lantero S, Sacco O, Rossi GA (1998). Downregulation of the expression of intercellular adhesion molecule (ICAM)-1 on bronchial epithelial cells by fenoterol, a β_2_-adrenoceptor agonist. J. Asthma.

[b31] Pan NY, Hui WS, Tipoe GL, Taylor GW, Leung RY, Lam WK (2006). Inhibition of pyocyanin-potenciated IL-8 release by steroids in bronchial epithelial cells. Respir. Med.

[b32] Papi A, Johnston SL (1999). Respiratory epithelial cell expression of vascular cell adhesion molecule-1 and its up-regulation by rhinovirus infection via NF-κB and GATA transcription factors. J. Biol. Chem.

[b33] Pauwels RA, Lofdahl CG, Postma DS, Tattersfield AE, O'Byne P, Barnes PJ (1997). Effects of inhaled formoterol and budesonide on exacerbations of asthma. N. Engl. J. Med.

[b34] Pérez L, Carrasco L (1993). Entry of poliovirus into cells does not require a low-pH step. J. Virol.

[b35] Pizzichini MMM, Pizzichini E, Efthimiadis A, Chauhan AJ, Johnston SL, Hussack P (1998). Asthma and natural colds. Inflammatory indices in induced sputum: a feasibility study. Am. J. Respir. Crit. Care Med.

[b36] Riise GC, Larsson S, Lofdahl CG, Andersson BA (1994). Circulating cell adhesion molecules in bronchial lavage and serum in COPD patients with chronic bronchitis. Eur. Respir. J.

[b37] Rogers DF, Barnes PJ (2006). Treatment of airway mucus hypersecretion. Ann. Med.

[b38] Ruff F, Zander JF, Edoute Y, Santais MC, Flavahan NA, Verbeuren TJ (1988). Beta-2 adrenergic responses to tulobuterol in airway smooth muscle, vascular smooth muscle and adrenergic nerves. J. Pharmacol. Exp. Ther.

[b39] Seemungal T, Harper-Owen R, Bhowmik A, Jeffries DJ, Wedzicha JA (2000). Detection of rhinovirus in induced sputum at exacerbation of chronic obstructive pulmonary disease. Eur. Respir. J.

[b40] Silvestri M, Fregonese L, Sabatini F, Dasic G, Rossi GA (2001). Fluticasone and salmeterol downregulate in vitro, fibroblast proliferation and ICAM-1 or H-CAM expression. Eur. Respir. J.

[b41] Skevaki CL, Christodoulou I, Spyridaki IS, Tiniakou I, Georgiou V, Xepapadaki P (2009). Budesonide and formoterol inhibit inflammatory mediator production by bronchial epithelial cells infected with rhinovirus. Clin. Exp. Allergy.

[b42] Subauste MC, Jacoby DB, Richards SM, Proud D (1995). Infection of a human respiratory epithelial cell line with rhinovirus. Induction of cytokine release and modulation of susceptibility to infection by cytokine exposure. J. Clin. Invest.

[b43] Suzuki T, Yamaya M, Sekizawa K, Yamada N, Nakayama K, Ishizuka S (2000). Effects of dexamethasone on rhinovirus infection in cultured human tracheal epithelial cells. Am. J. Physiol.

[b44] Suzuki T, Yamaya M, Sekizawa K, Hosoda M, Yamada N, Ishizuka S (2001). Bafilomycin A_1_ inhibits rhinovirus infection in cultured human tracheal epithelial cells: effects on endosomal pH and ICAM-1 production. Am. J. Physiol.

[b45] Suzuki T, Yamaya M, Sekizawa K, Hosoda M, Yamada N, Ishizuka S (2002). Erythromycin inhibits rhinovirus infection in cultured human tracheal epithelial cells. Am. J. Respir. Crit. Care Med.

[b46] Turner RB, Couch RB, Knipe DM, Howley PM (2006). Rhinoviruses. Fields virology.

[b47] Yamaya M, Nishimura H, Hatachi Y, Yoshida M, Fujiwara H, Asada M (2011). Procaterol inhibits rhinovirus infection in primary cultures of human tracheal epithelial cells. Eur. J. Pharmacol.

[b48] Yamaya M, Nishimura H, Hatachi Y, Yasuda H, Deng X, Sasaki T (2012). Inhibitory effects of tiotropium on rhinovirus infection in human airway epithelial cells. Eur. Respir. J.

[b49] Yasui K, Kobayashi N, Yamazaki T, Agematsu K, Matsuzaki S, Nakata S (2006). Differential effects of short-acting beta2-agonists on human granulocyte functions. Int. Arch. Allergy Immunol.

[b50] Yoshida N, Muraguchi M, Kamata M, Ikezono K, Mori T (2009). Procaterol potentiates the anti-inflammatory activity of budesonide on eosinophil adhesion to lung fibroblasts. Int. Arch. Allergy Immunol.

[b51] Zhu Z, Tang W, Ray A, Wu Y, Einarsson O, Landry ML (1996). Rhinovirus stimulation of interleukin-6 in vivo and in vitro. Evidence for nuclear factor κB-dependent transcriptional activation. J. Clin. Invest.

